# Persistent alveolar soft-part sarcoma with liver metastasis: a case report

**DOI:** 10.1186/1752-1947-4-233

**Published:** 2010-07-30

**Authors:** Olugbenga A Silas, Adeyi A Adoga, Agabus N Manasseh, Godwin O Echejoh, Raymond A Vhriterhire, Barnabas M Mandong

**Affiliations:** 1 Department of Pathology, Jos University Teaching Hospital, PMB 2076, Jos, Plateau State, Nigeria; 2 Department of Surgery, Jos University Teaching Hospital, PMB 2076, Jos, Plateau State, Nigeria

## Abstract

**Introduction:**

Alveolar soft-part sarcomas are rare, slow-growing tumors that metastasize commonly via vascular routes to the lungs, bones, lymph nodes and brain, causing morbidity and mortality. To the best of our knowledge, this is the first case describing metastasis to the liver reported from Nigeria.

**Case presentation:**

A 57-year-old man of the Urhobo ethnic group of Nigeria presented with a persistent mass in his left calf. It was initially diagnosed as soft-tissue sarcoma, and its associated systemic effects lead to his death before a histological diagnosis could be obtained.

**Conclusions:**

Alveolar soft-part sarcoma with metastasis to the liver can occur in our region (northeast Africa), and a high index of suspicion is required to make an early diagnosis, followed by prompt surgical excision with clear margins in order to prevent mortality.

## Introduction

Alveolar soft-part sarcoma (ASPS) is an extremely rare, vascular soft-tissue sarcoma affecting predominantly adolescents and young adults [1].

Accounting for less than 1% of soft tissue sarcomas, ASPS occurs commonly in the lower extremities (44%) and the head and neck (27%). Of the head and neck cases, 25% occur in the tongue [2] and show a predilection for women.

Because it has close clinical and imaging resemblance to common benign vascular tumors such as hemangioma, there is danger of misdiagnosis and, therefore, inadequate or delayed treatment. ASPS often involves the extremities of adolescents and young adults. Although the origin of ASPS is still unknown, recent cytogenetic studies revealed chromosomal rearrangements at t(X;17)(p11;q25) resulting in the ASPL-TFE3 fusion gene. Because women have an extra X-chromosome, their likelihood of developing an X autosomal translocation is theoretically double that of men. Thus, this extra X-chromosome is a possible explanation for the preponderance of ASPS in women [3,4].

Literature on ASPS is scant, especially in Nigeria and neighboring countries. In work done by Mandong *et al*. on the epidemiology of soft-tissue sarcomas in Jos, north central Nigeria, ASPS was found in only 3 (1.4%) of the 216 soft-tissue sarcomas studied over a period of 10 years [5].

ASPS is a slow growing tumor, which runs an indolent course with a poor prognosis, which metastasizes late to the lungs, bones, lymph nodes and brain [6,7]. Metastasis to the liver was reported in a large study of ASPS in China [8]. We therefore present the first case of ASPS with metastasis to the liver, with deleterious consequences, in our region.

Our case shows the fast metastatic potential of this tumor. Although it is most common among women and in the young to early middle-age group, it can occur in men into the sixth decade of life.

Because of the rapidity of metastasis with consequent mortality, a high index of suspicion is required to make a definitive diagnosis.

## Case presentation

A 57-year-old man of the Urhobo ethnic group of Nigeria presented to our hospital in January 2009 with an eight-month history of a persistent left calf swelling and one-week history of generalized body swelling. He had already undergone an excision of the swelling in another hospital five months prior to presentation, although no histologic report or statement on the margins of excision were available from this hospital. The swelling was recurred three weeks after the first excision and our patient sought help for his condition from traditional healers before presenting to our hospital.

The swelling was tender and he had difficulty moving his left leg. He was not diabetic or hypertensive. He had no other swelling.

Examination revealed a middle-aged man in painful distress with a solitary lymph node in the left inguinal region and non-pitting pedal edema in the affected limb. There was a fungating mass measuring 6×8 cm on the left calf. There was a wound with everted edges on the mass, which was bleeding on contact.

The surgeons made a provisional diagnosis of recurrent soft-tissue sarcoma. An abdominal ultrasound showed multiple hepatic metastases about 3 cm in greatest diameter. Random blood sugar (RBS) was 16.1 mmol/L, fasting blood sugar (FBS) was 10.6 mmol/L, and packed cell volume (PCV) was 27%. Chest and left leg X-rays were normal. Our patient could not afford a computed tomography (CT) scan.

He was admitted, transfused with a unit of blood and placed on subcutaneous soluble insulin, intravenous ciprofloxacin, metronidazole and ibuprofen tablets with daily wound dressing using Eusol.

One week later, the clinical condition of our patient deteriorated and he died following efforts at resuscitation. Surgeons delayed making a histological diagnosis. The reason given was that our patient presented with delirium and hyperglycemia, was thought to have diabetes mellitus and was unfit for excision biopsy. Therefore, the physicians were asked to review and metabolically stabilize our patient for surgery. The pathologists were asked to review him two days before his death, during which a biopsy of the leg swelling was taken for histology. Because of the rapidity of events leading to his death we were not able to obtain clinical photographs of the site of the lesion.

Five days after his death, a histology report was obtained as alveolar soft-part sarcoma with metastasis to the liver. Our patient's relatives declined an autopsy. Macroscopic features were a grayish-white tissue measuring 2×3 cm with overlying skin and a tiny grayish-white inguinal node biopsy tissue preserved in 10% formalin.

**Figure 1:**
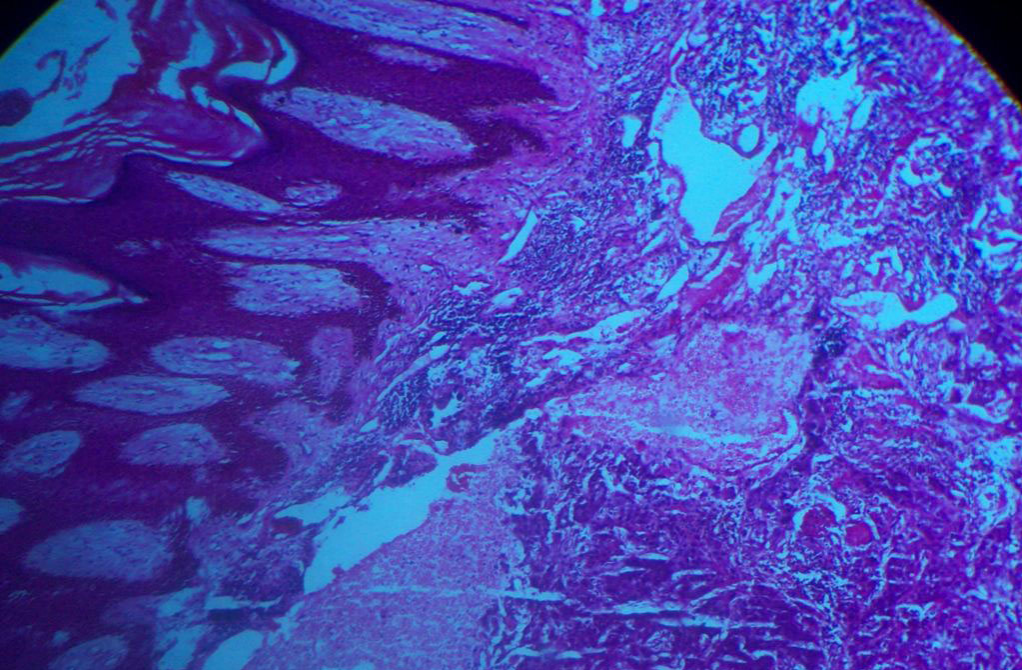
**Stratified squamous epithelium overlying a fibrocollagenous stroma in which neoplastic cells arranged in alveolar pattern are evident**.

**Figure 2:**
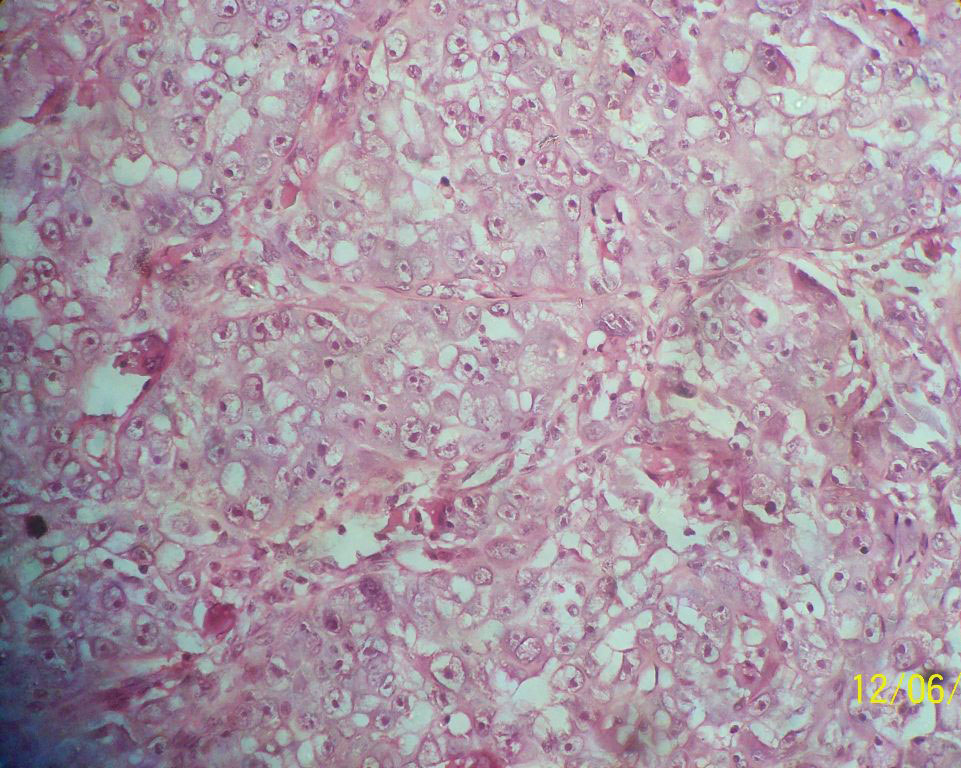
**Sheets of neoplastic cells exhibiting hyperchromasia, high nucleo-cytoplasmic ratio arranged in alveolar pattern by thin fibrous strands**.

Microscopy showed oval to polygonal cells with eosinophilic cytoplasm delineated by thin fibrovascular septae which separated the tumor cells into an organoid or nest-like pattern of arrangement with evidence of vascular invasion (Figures 1 and 2). These features are consistent with that of alveolar soft-part sarcoma.

## Discussion

Sarcomas are rare malignant tumors, arising from connective tissues and mostly found in the extremities [5], with ASPS being an extremely rare, vascular soft-tissue sarcoma affecting predominantly adolescents and young adults [1].

It was first described as a separate disease entity in 1952 [9]. Accounting for less than 1% of soft-tissue sarcomas, ASPS is commonly found in the lower extremities (44%) and in the head and neck (27%), with 25% of head and neck cases occurring in the tongue [2]. Women are more predisposed than men to ASPS. The peak age incidence among patients is 15 to 35 years. The pathogenesis of ASPS, as for most soft-tissue sarcomas, is unknown, although risk factors include irradiation, genetic factors and chemical carcinogens [2].

ASPS is a slow-growing tumor, which runs an indolent course with a poor prognosis, and metastasizes late to the lungs, bones lymph nodes and brain [6,7]. Reports of metastases to the liver were recorded in a large study in China [8]. The following lesions should be considered in the differential diagnosis of ASPS and they include paraganglioma, rhabdomyosarcoma, renal-cell carcinoma, metastatic adrenal carcinoma, clear-cell carcinoma and melanoma. Unlike paragangliomas, the cells are frequently discohesive and lack nuclear pleomorphism. Unlike alveolar RMS, the alveoli are not elongated and are not separated by fibrous tissue, and the cells are much larger than primitive myocytes. The tumor may closely resemble areas in renal-cell carcinoma, melanoma, metastatic adrenal carcinoma (often pleomorphic), and clear-cell sarcoma. The natural history of ASPS can be prolonged [10].

Histologically, ASPS is characterized by the presence of organoid nests of polygonal tumor cells encompassed by a dense capillary vasculature (Figures 1 and 2). The name "alveolar" was derived from its pseudo-alveolar appearance with clustered polygonal cells lacking central cohesion [11].

Recent cytogenetic studies show that ASPS exhibits a conserved abnormality in the form of an unbalanced translocation der(17)t(X;17)(p11;q25), which fuses the N-terminal region of the alveolar soft-part locus gene (*ASPL*), located at 17q25, to the C-terminal region of the transcription factor E3 (*TFE3*), located at Xp11. Two alternative fusion junctions have been observed resulting in the expression of two distinct fusion transcripts, *ASPL-TFE3 *type 1 and type 2 [2-4].

ASPS is a rare tumor. In a report on epidemiology of soft-tissue sarcoma in Jos, Nigeria, Mandong *et al*. observed that ASPS accounted for only 1.4% of total soft-tissue sarcomas diagnosed over a period of 10 years [5]. This is consistent with the low incidence recorded among Nigerian Igbos by Onuigbo [12].

Reports in other parts of the world have also show that ASPS is rare and of unknown histogenesis. Joyama *et al*. suggest that ASPS is a variant of rhabdomyosarcoma [13].

Our patient had metastases to the liver presenting with features of diabetes mellitus, the first such case reported in Nigeria.

Although spontaneous regression has been reported [14], surgical resection is the treatment of choice. However, the delay in making a definitive diagnosis led to our patient's death before he could benefit from this treatment. Diagnosis by histology is paramount and must be done immediately to institute definitive management, as ASPS has a high metastatic ability. A high index of suspicion is necessary and techniques ensuring fast histologic diagnosis, such as frozen section and immunohistochemistry for proper typing of tumors, are important. Unfortunately, our center does not have these facilities.

Our case shows the ignorance of some health practitioners, especially those in peripheral hospitals, about the need for histologic reports of any mass excised from a patient. Medical doctors in the developing world must inculcate evidence-based medicine in the management of patients. The role of the pathologist in patient management cannot be overemphasized as the incidence of cancers gradually increases worldwide, including Africa. Although radiologic diagnosis maybe helpful, it may be limited in the typing of tumors for effective treatment. Hospitals should therefore be equipped with histologic and cytologic diagnostic tools for early detection of malignancies and to reduce morbidity and mortality.

## Conclusions

ASPS with metastasis to the liver does occur in Nigeria. A high index of suspicion is required to make an early diagnosis. After this, a prompt surgical excision with clear margins should be done in order to avoid fatal outcomes.

## Consent

Written and informed consent for the publication of this report and any accompanying images was obtained from the daughter of our patient. A copy of the written consent is available for review by the Editor-in-Chief of this journal.

## Competing interests

The authors declare that they have no competing interests.

## Authors' contributions

OAS conceived the report and prepared the histologic slides and manuscript. AAA performed the literature search and prepared the manuscript. ANM read the slides. GOE reviewed the final manuscript. ARV performed the literature search. BMM reviewed the final manuscript. All authors have read and approved the final manuscript.
